# The practical use of genome sequencing data in the management of a feline colony pedigree

**DOI:** 10.1186/s12917-017-1144-y

**Published:** 2017-07-27

**Authors:** Fabiana H. G. Farias, Chad Tomlinson, Jeffrey Labuda, Gerardo Perez-Camargo, Rondo Middleton, Wesley C. Warren

**Affiliations:** 1 McDonnell Genome Institute, Washington University School of Medicine, Saint Louis, MO 63108 USA; 2 Nestlé Purina Research, Saint Louis, MO 63164 USA

**Keywords:** Whole-genome sequencing, Cats, Loss of function variants

## Abstract

**Background:**

A higher prevalence of inherited disorders among companion animals are often rooted in their historical restricted artificial selection for a variety of observed phenotypes that eventually decreased genetic diversity. Cats have been afflicted with many inherited diseases due to domestication and intense breed selection. Advances in sequencing technology have generated a more comprehensive way to access genetic information from an individual, allowing identification of putative disease-causing variants and in practice a means to avoid their spread and thus better pedigree management. We examine variants in three domestic shorthair cats and then calculated overall genetic diversity to extrapolate the benefits of this data for breeding programs within a feline colony.

**Results:**

We generated whole genome sequence (WGS) data for three related cats that belong to a large feline pedigree colony. Genome-wide coverage ranged from 27-32X, from which we identified 18 million variants in total. Previously known disease-causing variants were screened in our cats, but none carry any of these known disease alleles. Loss of function (LoF) variants, that are in genes associated with a detrimental phenotype in human or mice were chosen for further evaluation on the comparative impact inferred. A set of LoF variants were observed in four genes, each with predicted detrimental phenotypes as a result. However, none of our cats displayed the expected disease phenotypes. Inbreeding coefficients and runs of homozygosity were also evaluated as a measure of genetic diversity. We find low inbreeding coefficients and total runs of homozygosity, thus suggesting pedigree management of genetic relatedness is acceptable.

**Conclusions:**

The use of WGS of a small sampling among a large feline colony has enabled us to identify possible disease-causing variants, their genotype state and measure pedigree management of genetic diversity. We contend a limited but strategic sampling of feline colony individuals using WGS can inform veterinarians of future health anomalies and guide breeding practices to ensure healthy genetic diversity.

**Electronic supplementary material:**

The online version of this article (doi:10.1186/s12917-017-1144-y) contains supplementary material, which is available to authorized users.

## Background

The use of pedigreed colonies remains a powerful resource to study many phenotypes of interest in great detail, yet among companion animals, specifically dogs and cats, large well-maintained pedigrees for such use are rare and not readily available. Given this rarity, their optimal use in the understanding of health and behavioral well-being is of crucial importance. Disease surveillance is a critical component of comprehensive veterinary care programs to detect and prevent the spread of disease within animal colonies, thereby enhancing the quality of life of these animals. Veterinary health checks routinely include the collection of samples that can provide a means to detect existing or future health problems and thus provide appropriate care directly to benefit the animal. With the advances underway in the collection of electronic medical records for companion animal patients, mimicking efforts in human clinical practice, the ability to return to banked samples for basic disease research or clinical testing to provide optimal care has veterinarians excited about these health management opportunities. Data collection such as a whole genome or targeted sequencing, immunoassays, metabolite profiling, fixed genotyping and others, collectively or in isolation can drive discovery of the sources of trait diversity linked to genetic variation.

Information provided by genetic data can aid breeding programs by reducing introduction and propagation of health problems in a pedigree. There are many diseases in animals that have been associated with gene variants, for some of those variants, there are commercial genetic tests available [1]. The ability to select individuals based on genetic information circumvents the issue of producing progeny with health issues that can be unfavorable for these animals. Especially in occasions where disease symptoms appear later than breeding age, without genetic information, those animals will be included in the breeding program resulting in dissemination of undesirable traits. Polycystic kidney disease (PKD) in Persian cats is an example of a disease in which symptoms appear after breeding age [2]. Lyons et al. [3] have identified the causative mutation for PKD in the gene *PKD1*, and a commercial genetic test is available enabling Persian cat breeders to make mating decisions based on genetic information.

WGS although still costly for companion animal veterinary practice is the most comprehensive method for detection of an individual’s genetic variation. WGS has enabled enormous progress in understanding disease in human and animals. Moreover, it allows extensive evaluation of genetic diversity which is essential for the maintenance of a healthy pedigree. However, the interpretation of the enormous amount of genetic information generated remains a difficult task and reference assembly quality for the cat presents additional variant detection challenges [4]. The cat has a reference genome that was first assembled with a 1.9X coverage genome sequence of an inbred Abyssinian cat [5]. Additional sequencing of the same cat to 14X and other cat breeds have allowed enhancement of reference and the identification of common variation in the cat genome [6]. However, the cat reference still has flaws, such as gaps and unplaced sequences (not in chromosomal regions) and these problems often hinder the discovery of variants associated with phenotypes.

Genetic variant interpretation has been a massive challenge in genetic studies of any species. However, large-scale human disease cohort sequencing projects and even more important the development of databases containing common variation and variants associated with disease have eased this burden of following false positive candidates. In dogs and cats, there have been variants deposited into databases, such as dbSNP, but there is no information on frequency, breed or health status of the individuals from which variants were discovered. Without such data, future efforts to associate putatively damaging variants with disease outcome are much less efficient.

In this preliminary study of a feline colony pedigree, we generated WGS data from three related cats within the pedigree, which contains historical data from ~800 cats, in order to survey segregation of potential disease variants and genetic diversity. We evaluated single nucleotide variants (SNVs) and then compared to databases containing information on genes associated with a disease. Additionally, we calculated runs of homozygosity and inbreeding coefficients on each as a measure of genetic health.

## Methods

### Animal descriptions

Three cats were selected for WGS that are part of a pedigreed population maintained by Nestlé Purina as a resource to study behavior preferences and nutritional developments. The pedigree consists of Domestic shorthair cats. We selected cats that were placed at an intermediate position in the total pedigree structure. Also, these cats were directly related allowing observation of accumulation of damaging variants and if there is a decrease in genetic diversity. The health of the cat colony is provided by a veterinary team with a proactive attitude towards disease management. All cats have regular health screening tests depending of their age risk and individual cases. Veterinary care is provided in the same manner and principles than to any individually owned house cat visiting a veterinary clinic, and all individual clinical histories are recorded.

**Fig. 1:**
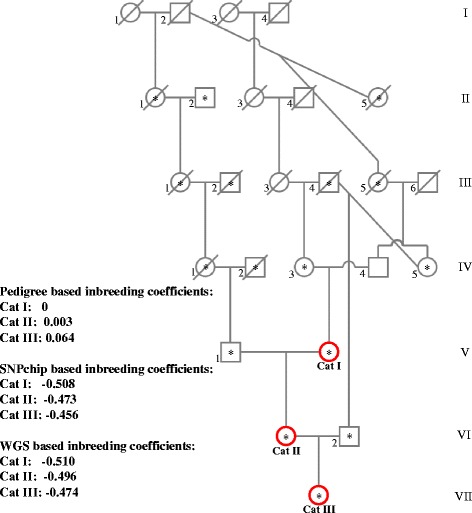
Pedigree and inbreeding coefficients. The pedigree is showing close related individuals to the three WGS cats (red circles). Squares represent males and circles represent females. Diagonal lines across symbols represent deceased cats. The asterisk indicates cats that have SNPchip data. On the left are the inbreeding coefficients calculated based on pedigree, SNPchip and WGS data

An example of an extended family within this pedigree is depicted in Fig. 1, including the three WGS cats (Cat I, Cat II, and Cat III). Cat I is an 8-year-old female that is the dam of Cat II and has eight other offspring in the pedigree. Cat II is a 6-year-old female that has about 40 half siblings on the pedigree. Also, Cat II is the dam of Cat III and has two other offspring. Cat III is a 5-year-old female with no offspring, but it has 15 half siblings in the pedigree. All three cats were healthy based on annual veterinarian physicals and no observed disease symptoms during routine care. Samples of whole blood were collected from each cat, by trained veterinary staff, into an Acid Citrate Dextrose vacutainer tube. DNA was isolated using the MagNA Pure 96 (Roche Diagnostics) automated instrument according to the manufacturer’s instructions.

### Whole-genome sequencing and variant detection

Cats were sequenced on an Illumina HiSeq X10 instrument with 350 to 550 bp PCR-free libraries to 150 bp read length. The sequence data for each cat were aligned to the chromosomes of the domestic cat reference assembly (*Felis catus* 8.0) using Speedseq [7]. Variants, specifically SNVs and small indels (<10 bp), were called using The Genome Analysis Toolkit (GATK) HaplotypeCaller and GenotypeGVCFs [8]. Samtools flagstat [9] and GATK DepthofCoverage were used to extract sequence alignment statistics. SNVs were then extracted from all variants for further evaluation. All SNVs were annotated using the Variant Annotation, Analysis and Search Tool (VAAST 2) [10]. Next, SNVs predicted to severely disrupt protein-coding genes, loss of function (LoF) variants, shared between individuals or unique to each were reported. In this study, we only considered LoF variants with the most likely deleterious impact: splice sites disruption, stop gain or loss, and frameshifts.

### Variant validation and genotyping

To estimate our number of false SNVs detected, we selected five variants to be validated by Sanger sequencing in all three cats. We found two were homozygous, and three were heterozygous genotypes. Additionally, two closely related cats and five unrelated cats from the pedigree were genotyped for the same variants to evaluate expected genotypes. Sequences were amplified by PCR using specific primers (Additional file 1: Table S1) and Amplitaq Gold polymerase kit (Thermo Scientific) according to the manufacturer’s protocol with the following modifications: AmpliTaq Gold concentration was 2.5 U/reaction, the forward and reverse primer concentrations were 0.4 μM (final concentration), and the denature time was 30 s. PCR products were purified using PureLink PCR purification kit (Thermo Scientific) following manufacturer’s protocol. After purification PCR products were sent to GeneWiz (South Plainfield, NJ) for Sanger sequencing. In addition, a set of 20 heterozygous SNVs (Additional file 1: Table S2) genotyped on the Illumina feline 63 K SNP BeadChip [4] from the same three WGS cats were validated for equivalency of SNVs calls.

### Detection of variant impact

The databases Online Mendelian Inheritance in Man (OMIM) [11], Online Mendelian Inheritance in Animals (OMIA) [12] and the database of essential genes (DEG) [13] were consulted for information on disease-causing genes. We only considered genes in OMIM that were associated with a phenotype and in OMIA, only genes that were associated with phenotypes in cats. Also, genes considered essential for mice were incorporated from DEG in the analysis. All LoF variants in disease-causing genes were manually evaluated with the Integrative Genomics Viewer (IGV) [14], and SNV effect on each gene was assessed by comparison of protein translation to other mammals with NCBI blastp using default parameter settings.

### Known disease variants screening

Variants associated with disease in cats that have a commercial DNA test available were screened in our three cats by inspection of genotypes or presence of deletions for the specific positions on sequence data. The list of variants screened is in Additional file 1: Table S3 with respective positions on cat reference version 8.0.

### Inbreeding coefficient estimation

Pedigree-based inbreeding coefficient (IC) was calculated using the Wright’s equation:

$$ {F}_X=\sum \left[{\left(\frac{1}{2}\right)}^{n_1+{n}_2+1}\left(1+{F}_A\right)\right] $$

Where *F*_*x*_ is the inbreeding coefficient of the cat in question, *F*_*A*_ is the inbreeding coefficient of the common ancestor, *n*_1_ is the number of generations from the sire to the common ancestor, and *n*_2_ is the number of generations from the dam to the common ancestor. We used the known information about the pedigree (Fig. 1), however, there were unknown ancestors. IC was calculated for Cat I based on three generations, Cat II based on five generations and Cat III based on 6 generations.

Genotype data from the Illumina feline 63 K SNP BeadChip (SNPchip) and WGS data based inbreeding coefficients were calculated using PLINK v1.07 [15] --het function. The SNPchip data was analyzed with 55,053 SNPs, while the WGS data was analyzed with 13,455,757 SNPs, both from autosomes only. Since we have SNPchip data from 297 cats that are part of the pedigree, we calculated IC using data for all the cats to compare scores when calculating with only the three cats.

### Runs of Homozygosity analysis

To calculate runs of homozygosity (RoH) for SNPchip and WGS data we used PLINK v1.07 --homozyg function. For SNPchip data, we defined our RoH segments as five or more consecutive homozygous SNVs per individual. For WGS data we used a window size of 250 kb since this approximate window size is roughly equivalent to five homozygous SNVs on SNPchip data.

## Results

### Whole genome sequencing and variant validation

We generated WGS for each cat with an average range of 27-32X coverage. The number of sequences that properly mapped to Felis_catus-8.0 reference was between 92 and 97%, and duplicates were between 13 and 15% (Table 1). A total of 18,137,177 variants were identified in all three cats, 14,088,779 were SNVs and 4,048,398 were indels (Table 2). For this study we only report putatively deleterious SNVs since indels are known to have high rates of false positives [16].

**Table 1:** Whole-genome sequencing results summary

	Cat I	Cat II	Cat III
Number of reads	639,766,569	575,104,522	552,808,448
Average coverage	32.13	27.86	27.79
Duplicates	15.52%	13.76%	14.78%
Mapped reads	97.19%	92.83%	96.70%
Properly paired	94.84%	89.99%	93.96%

**Table 2:** The number of variants identified for each cat, including SNPs and Indels. Variants are divided by annotation categories

	Total	Non-synonymous	Frameshift	Splice site	Stop gain/loss
Cat I					
all	13,791,282	28,984	2394	656	650
homozygous	4,722,225	8502	1106	237	179
unique	1,947,067	4256	290	104	110
Cat II					
all	13,663,921	28,961	2414	636	627
homozygous	4,808,547	8605	1115	255	170
unique	1,142,035	2399	244	70	58
Cat III					
all	13,635,215	28,467	2380	632	622
homozygous	4,882,667	8869	1115	230	189
unique	1,708,602	3399	317	92	79
Shared					
all	9,196,149	19,377	1730	434	407
homozygous	2,597,948	4947	898	131	108

We selected five SNVs (Additional file 1: Table S1) for validation and genotyping in seven additional cats. The two homozygous SNVs (chrE1:40,235,385 and chrE1:40,235,189) were validated and also present on the additional seven cats. The three heterozygous SNVs were not validated, all cats were homozygous for the reference allele. It was surprising that all three heterozygous SNVs were false positives. Examination of the regions containing the SNVs revealed that two of those regions were located within sequences homologous to other sequences in the cat genome, which may suggest that misalignment created the false positives. The convergence between SNPchip heterozygous genotypes and WGS SNP showed 100% agreement between calls, indicating our SNV calls are of high confidence for further study.

### Variant functional evaluation

**Fig. 2:**
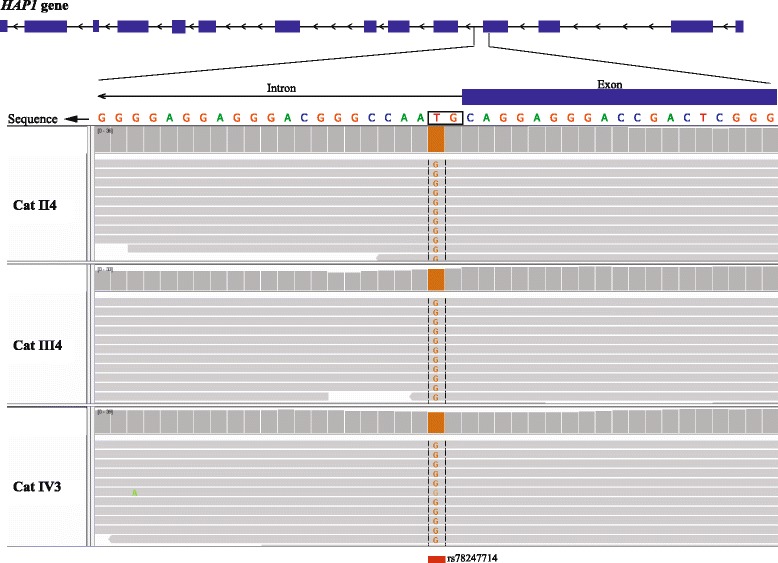
Homozygous LoF on *HAP1* gene. The variant in *HAP1* gene is homozygous on all three cats and it changes the splice donor sequence from GT to GG. This variant has been previously identified in other cats and has been deposited in dbSNP

Genes that harbored LoF variants were cross-referenced with three databases containing information on phenotypes associated with genes: OMIM, OMIA, and DEG. Next, we manually checked variants in IGV and the protein translation similarity to other mammals. From the genes associated with a phenotype, there was one homozygous and two heterozygous shared LoFs between the three cats, while there was one heterozygous unique LoF. We first investigated the LoF variants that were shared between the three cats in a homozygous and heterozygous state. Only one homozygous LoF was identified in a gene that matched OMIM and DEG databases; it is an SNV that changes the splice site sequence on the huntingtin-associated protein 1 (*HAP1*) gene (Fig. 2). *AHR* and *CTNNA2*, which are genes in the DEG database, both have a heterozygous LoF predicted to disrupt splice sites. Furthermore, we investigated unique LoFs homozygous and heterozygous in each cat and no unique homozygous LoFs in genes that matched the databases were found. There was only one heterozygous LoF in Cat III that creates a stop five amino acids before the end of the immunoglobulin mu binding protein 2 (*IGHMBP2*) protein. The positions and types of LoFs are described in Additional file 1: Table S4. Overall, we observed a high number of false positive LoFs at ~42%, based on the variants manually inspected, that we attribute to inaccurate gene models.

### Screening for known cat disease variants

To ascertain if our screening would find known disease alleles, even though our cats were deemed healthy by veterinarian exams, we screened our three cats for variants that match these causative alleles. Each has accompanying commercial DNA tests that could confirm their putative disease carrier status in our pedigree. Some of these diseases are breed specific while all cats that are part of this pedigree are mixed breed so their frequency would be expected to be rare and in some cases may not present disease phenotype in a mixed genetic background. The diseases screened for relevant variants were: Gangliosidosis 1 [17], Gangliosidosis 2 [18], Cardiomyopathy [19, 20], Hypokalemia [21], Progressive retinal atrophy [22], Polycystic kidney [3], and Spinal muscular atrophy [23]. The occurrences of some of these diseases in our pedigree lead us to screen these variants even though the three cats selected are healthy. We found no instances matching disease variants.

### Inbreeding coefficients

We calculated IC to gauge genetic diversity using available pedigree relationships, SNPchip and WGS data for all three cats (Fig. 1 **)**. New male cats were frequently introduced to the pedigree to keep genetic diversity high but in most cases, these sires lack ancestry information as shown in Fig. 1. Pedigree based IC was calculated on available information on ancestors using the Wright’s equation. Cat III had the highest pedigree based IC, most likely due to this cat having more pedigree information than the others. IC from SNPchip and WGS data were calculated on the observed versus expected number of homozygous genotypes. SNPchip and WGS based ICs were equivalent, as is expected since both were calculated by the same method. The IC was remarkably low in all three analyses. Since there is missing ancestry information for some of the cats, IC calculated based on SNPchip and WGS data are more reliable than the pedigree-based. However, IC calculated by PLINK is more accurate when calculated with larger sample size. When IC was calculated with 297 individuals the scores were higher (Aditional file 1: Table S5) while still negative indicating high rate of heterozygosity (CatI: −0.508, −0.057; CatII: −0.473, −0.034; CatIII: −0.456, −0.022; IC calculated with three cats and IC calculated with 297 cats respectively).

### Detection of RoH in SNPchip and WGS data

The estimation of the level of homozygosity in our pedigree associated cats was carried out with SNPchip and WGS data using different window sizes, five consecutive homozygous SNPs and 250 kb, respectively. WGS analysis was done in window size instead of a number of consecutive homozygous SNPs, because the distances between SNPs are on average less than 500 bp apart compared to SNPchip markers that are on average 50 kb apart. SNPchip data analysis detected 20–27 RoH segments (Additional file 1: Table S6) while WGS data analysis detected 24–57 RoH segments (Additional file 1: Table S7). The higher number of RoH identified by WGS data is expected given the higher resolution of detected variants. Comparison of the analysis of the two datasets shows that there was considerable overlap for both, but the length of the RoH was much higher for SNPchip data (average 6344 kb SNPchip, average 324 kb WGS). RoH segments identified by SNPchip data were frequently broken into smaller RoH segments identified by WGS when there was overlap. Some of the segments are fully or partially shared between cats for each data set. Cat III has the higher number of RoH for both data sets (27 for SNPchip and 57 for WGS) compared to the other two cats. The low numbers of RoH segments identified corresponds to the low IC observed for theses cats calculated with SNPchip and WGS data.

### Discussion

Genetic diversity plays an important role in maintaining a healthy pedigree. While the success of a genetically diverse pedigree relies on an effective breeding program, mating decisions with inadequate genetic information could have unintended consequences in future generations. The traditional process of selecting individuals for breeding involves calculations of inbreeding prior to mating in order to optimize hybrid vigor as well as consideration of traits and symptoms when they became apparent in the sire or dam and offspring. Unlike the mating decisions made in food-producing animals, determined largely by the need to improve phenotypes of economic interest, health is the major priority in managing companion animal pedigrees. The identification of genetic variants within genes implicated in clinically relevant phenotypes provides a new means to avoid the spread of unintended alleles with harmful outcomes before breeding. Already veterinarians are attempting to utilize genetic information as a diagnostic and clinical management aid. However, limited validation of numerous putative alleles of clinical significance has hampered their abilities. In a well-maintained pedigree, undesirable recessive phenotypes can be avoided by selective breeding to circumvent the production of homozygous individuals, thus preventing propagation of individuals with potentially adverse health conditions. We contend that the use of WGS data to assess sire and dam mutational profiles and to determine genetic diversity can help to improve animal health and in maintaining offspring genome diversity in the pedigree.

In our limited study of pedigree associated cats, we first analyzed potential clinical relevant variants. Several variants have been associated with diseases in cats [1] and some have commercially available genetic tests for breeders to screen their animals. Nonetheless, not all tests are applicable breed wide, for example, the Gangliosidosis 2 *HEXB* variant [18] is specific for the Burmese breed. The mixed breed cats in our pedigree do show occurrences of a few of these diseases in the pedigree, such as Polycystic kidney disease (PKD). PKD was estimated to have a prevalence of 30–38% worldwide in Persian and closely related cat breeds [24–26]. Therefore, there was a possibility that the cats in our pedigree carry one or more of the PKD disease alleles. However, screening for all known disease variants revealed that our cats do not carry any of these causative alleles, but continued surveillance is needed for the appearance of new disease causative alleles.

The tremendous expansion of variant knowledge among human studies can reveal shared genic events, at least within the same gene, that may be of clinical relevance in veterinarian care. In this report, we find several cases of shared variants that could lead to future health consequences but most often is undetected phenotypically. Shared among the three cats we have identified a homozygous splice-site SNV in the gene *HAP1*. This SNV has been identified previously in other cats according to dbSNP, rs784247714, and it was also identified in the additional seven cats we genotyped from the pedigree. The HAP1 protein interacts with the huntingtin protein [27], which is associated with Huntington disease [28]. However, *HAP1* itself has not been directly linked to Huntington disease. Chan et al. [29] have shown that *Hap1* knockout mice exhibit strikingly depressed feeding behavior and are unable to gain body weight after birth. The mice often die after day 2–3, but the ones that survive displayed growth retardation with apparent normal brain and behavioral development suggesting an effect only in early postnatal feeding behavior [30]. In our three cats, no similar abnormal feeding behavior has been observed, which may suggest that this mutation does not affect the protein function. Alternatively, the creation of a protein isoform that skips one exon is fully functional.

We identified two heterozygous LoF variants shared by the three cats that matched *AHR* and *CTNNA2*, which are considered essential genes for survival in mice according to DEG. The aryl hydrocarbon receptor plays important roles in the developmental remodeling of vascular architecture in the liver [31], regulates the toxicity of halogenated dioxins [32] and controls the adaptive up-regulation of xenobiotic metabolizing enzymes in response to polycyclic aromatic hydrocarbons [33]. In *Ahr*-null mice disruption of AHR signaling pathway causes fetal necrosis and consequent liver deformation which persists through adulthood [34]. The CTNNA2 protein links the classical cadherins to the neuronal cytoskeleton and is expressed only in the central nervous system in mice [35]. Mice lacking part of the CTNNA2 protein are ataxic and show abnormal lobulation of the cerebellum and cerebellar hypoplasia [36]. These phenotypes haven’t been observed in the pedigree so far, however, our discoveries highlight how pedigree breeding management would provide a means to avoid the propagation of these alleles in subsequent generations.

Additionally, we explored unique LoFs for each cat. We identified a heterozygous stop gained SNV on *IGHMBP2* gene unique to Cat III. Mutations in this gene are reported to cause distal spinal muscle atrophy type 1 [37] and Charcot-Marie-Tooth disease type 2 [38]. The stop gained, found in our cat, was 5 amino acids before the end of the protein, it is most likely that this SNV do not affect protein function. Given its haploid state in Cat III and no observed health abnormalities this variant is not considered to be a risk for disease development, therefore not affecting the decision to include this cat in the breeding pool. The effect of variants in gene function is not easily predicted. Despite the tools that classify variants as benign or damaging, it is of substantial advantage to having access to a common variants database, where information on health status and breed are recorded for each variant identified. In this example, we would greatly benefit from that information to determine if this SNV is benign or damaging. The need for a robust repository for variants in cats is critical for research in disease or trait variant discovery. In humans, great efforts have been made to create databases recoding variant information with different levels of evidence implicating variants in disease risk or causation, such as ClinVar. Also, guidelines for associating variants to disease have been described to avoid a proliferation of false positive findings [39].

To access inbreeding status of the three cats, IC was calculated based in three data sets: pedigree information, SNPchip, and WGS data. Pedigree information was limited for part of the ancestors because cats are frequently introduced to the pedigree. ICs based on pedigree were higher than the ones calculated with SNPchip and WGS data. SNPchip and WGS ICs were interchangeable for each of the cats; the ICs were negative indicating a high rate of heterozygosity relative to their reference population. However, we observed that larger sample sizes are recommended for higher accuracy when calculating IC with genetic data. Our results for a small sampling of the pedigree reveal efforts to maintain diversity is successful thus far. The analysis of runs of homozygosity (RoH) with SNPchip and WGS data has shown that SNPchip analysis overestimates length and underestimates the number of RoH. WGS has a better resolution for this type of analysis because it genotypes every position while SNPchip analysis doesn’t consider heterozygosity between markers and markers are in average 50 kb apart. The number of RoH segments identified for both datasets are in agreement with the low ICs observed.

## Conclusions

In summary, we describe the assessment and possible use of genomic information of three cats from a large pedigree. We are cognizant of the limitations that three cat genomes could provide to the management of large pedigrees, nonetheless, our genome variant data has enabled us to identify possible disease causing variants, plan more cost-effective screening assays using this data and obtain an estimate of pedigree genetic diversity. The decision on inclusion and exclusion of cats in the breeding pool based on genetic variants must be carefully considered. First and foremost, variants that are known to cause disease should be regarded as most important during breeding management. The variants predicted to be damaging should be complemented by clinical observations of animals to conclude that they impact health. Since genetic diversity is as crucial as avoiding the spreading of disease-causing variants, it is necessary to balance the breeding pool via mating selections that safeguard genetic diversity while minimizing the accumulation of damaging variants. Once disease variants are discovered, they can be cost effectively screened as part of marker panels, much akin to human clinical disease screening protocols, to better manage pedigree health. The expectation is genome data strategically collected can be a powerful tool to improve animal health.

## Additional file

Additional file 1: Additional tables. **Table S1.** Validation primer sequences and PCR annealing temperatures; **Table S2.** Heterozygous SNPs selected from SNPchip data for cross validation; **Table S3.** Variants associated with diseases in cats with a commercial DNA test available; **Table S4.** LoF variants identified in genes associated with disease; **Table S5.** Inbreeding coefficient calculated with SNPchip data of only 3 cats and with SNPchip data of 297 cats ; **Table S6.** RoH identified with SNPchip data on the three cats; **Table S7.** RoH identified with WGS data on the three cats. The last column has the overlap with SNPchip RoH number. (DOCX 42 kb)

## Abbreviations

DEG: Database of Essential Genes
IC: Inbreeding Coefficient
IGV: Integrative Genomics Viewer
LoF: loss of function
OMIA: Online Mendelian Inheritance in Animals
OMIM: Online Mendelian Inheritance in Man
PKD: Polycystic kidney disease
RoH: Runs of Homozygosity.
SNV: Single nucleotide variant
WGS: Whole genome sequence
