# Supplementary material for: The practical use of genome sequencing data in the management of a feline colony pedigree
Source: BMC Vet Res. 2017 Jul 27;13:225. doi: 10.1186/s12917-017-1144-y (PMC5532773; doi:10.1186/s12917-017-1144-y)
Supplement: Additional file 1: — Additional tables. Table S1. Validation primer sequences and PCR annealing temperatures; Table S2. Heterozygous SNPs selected from SNPchip data for cross validation; Table S3. Variants associated with diseases in cats with a commercial DNA test available; Table S4. LoF variants identified in genes associated with disease; Table S5. Inbreeding coefficient calculated with SNPchip data of only 3 cats and with SNPchip data of 297 cats ; Table S6. RoH identified with SNPchip data on the three cats; Table S7. RoH identified with WGS data on the three cats. The last column has the overlap with SNPchip RoH number. (DOCX 42 kb) [file 12917_2017_1144_MOESM1_ESM.docx]

**Additional file 1: Additional tables**

T**able 1.** Validation primer sequences and PCR annealing temperatures.

| SNP position |  | Primer sequences | Annealing temp. |
| --- | --- | --- | --- |
| chrB3:135375263 | forward | GAAACGGAGAGATTAGTGAAAGG | 55.8ºC |
|  | reverse | GCTTCTTTGGCAGCAACTATATC |  |
| chrB4:25318732 | forward | GCAATCCCTAACTTTGGTTTAG | 51.9 ºC |
|  | reverse | GTATCTATCATCCAAACAATGG |  |
| chrE1:40235385 and chrE1:40235189 | forward | GGACCTAAATACTGCGGCTCG | 56.3 ºC |
|  | reverse | CGTCAGTATTCCTGTTTGCAC |  |
| chrE3:39434301 | forward | GAAGATATAATTGACACTGGC | 51.5 ºC |
|  | reverse | GCAGCTGGCCACAGATGTAG |  |

**Table 2.** Heterozygous SNPs selected from SNPchip data for cross validation.

| **SNP name** | **Chromosome*** | **Position*** |
| --- | --- | --- |
| chrA1.10372133 | chrA1 | 8521613 |
| chrA1.118784743 | chrA1 | 94075620 |
| chrA2.121781900 | chrA2 | 70816919 |
| chrA3.131987714 | chrA3 | 102454466 |
| chrB1.138386362 | chrB1 | 112808095 |
| chrB2.91073640 | chrB2 | 71815641 |
| chrB3.95256323 | chrB3 | 87295470 |
| chrB4.120917733 | chrB4 | 103353275 |
| chrC1.180251354 | chrC1 | 161540704 |
| chrC2.119409898 | chrC2 | 104344104 |
| chrD1.7813859 | chrD1 | 6702627 |
| chrD2.104655560 | chrD2 | 77334907 |
| chrD3.119526396 | chrD3 | 85778841 |
| chrD4.19862348 | chrD4 | 23777630 |
| chrE1.70369926 | chrE1 | 37871040 |
| chrE2.7411556 | chrE2 | 5133321 |
| chrE3.59560093 | chrE3 | 31335355 |
| chrF1.39935522 | chrF1 | 34306320 |
| chrF2.17454415 | chrF2 | 15137840 |
| chrUn.65020536 | chrX | 652223 |

*Position based on cat reference 8.0

**Table 3.** Variants associated with diseases in cats with a commercial DNA test available.

| **Disease** | **Gene** | **Chr** | **Mutation** | **Ref. v8.0 pos** |
| --- | --- | --- | --- | --- |
| Gangliosidosis 1 | GLB1 | C2 | Arg483Pro | C2:157031480 |
| Gangliosidosis 2 | HEXB | A1 | c.1356_1362delGTTCTCA | A1:139630550-139630563 |
| Cardiomyopathy | MYBPC3 | D1 | A31P | D1:99555575 |
| Cardiomyopathy | MYBPC3 | D1 | R820W | D1:99544451 |
| Hypokalemia | WNK4 | E1 | Q968X | E1:41100504 |
| Progressive retinal atrophy | CEP290 | B4 | IVS50 + 9T>G | B4:110438888 |
| Polycystic kidney disease | PKD1 | E3 | C3288X | E3:39439369 |
| Spinal muscular atrophy | LIX1-LNPEP | A1 | 140k deletion | A1:159200000-159340000 |

**Table 4.** LoF variants identified in genes associated with disease.

| Variant | Gene | Reference | Alternative | LoF type | Position |
| --- | --- | --- | --- | --- | --- |
| Shared hom | *HAP1* | A | G | splice site | chrE1:40235385 |
| Shared het | *AHR* | G | A | splice site | chr:A2:109163741 |
| Shared het | *CTNNA2* | G | A | splice site | chrA3:95477515 |
| Unique Cat III | *IGHMBP2* | G | A | stop gained | chrD1:111186703 |

**Table 5.** Inbreeding coefficient calculated with SNPchip data of only 3 cats and with SNPchip data of 297 cats.

| **Cats** | **IC calculated only with 3 cats** | **IC calculated with all 297 cats** |
| --- | --- | --- |
| **Cat I** | -0.508 | -0.057 |
| **Cat II** | -0.473 | -0.034 |
| **Cat III** | -0.456 | -0.022 |

**Table 6.** RoH identified with SNPchip data on the three cats

|  | **Chr** | **Start pos.** ^*^ | **End pos.** ^*^ | **Length in KB** | **# of SNPs** | **RoH #** |
| --- | --- | --- | --- | --- | --- | --- |
| **Cat I** | A1 | 12974208 | 15322524 | 2348 | 69 | 1 |
|  | A1 | 113915036 | 115803218 | 1888 | 55 | 2 |
|  | A2 | 98710797 | 101675099 | 2964 | 68 | 3 |
|  | A3 | 31131207 | 36448204 | 5317 | 138 | 4 |
|  | A3 | 94912125 | 115291118 | 20379 | 54 | 5 |
|  | B1 | 41557458 | 44812023 | 3255 | 79 | 6 |
|  | B1 | 147431217 | 157094532 | 9663 | 231 | 7 |
|  | B1 | 157327126 | 162150350 | 4823 | 105 | 8 |
|  | B3 | 111732052 | 114090366 | 2358 | 57 | 9 |
|  | B4 | 21236215 | 27309184 | 6073 | 164 | 10 |
|  | B4 | 71709760 | 73816119 | 2106 | 50 | 11 |
|  | B4 | 105973535 | 108011392 | 2038 | 52 | 12 |
|  | C1 | 74351167 | 95114175 | 20763 | 507 | 13 |
|  | C1 | 91568052 | 114437430 | 22869 | 157 | 14 |
|  | C1 | 194960094 | 197659658 | 2700 | 74 | 15 |
|  | C2 | 54946161 | 68256349 | 13310 | 52 | 16 |
|  | D2 | 16375919 | 17500147 | 1124 | 35 | 17 |
|  | D2 | 47183307 | 49478651 | 2295 | 52 | 18 |
|  | D2 | 62661535 | 64913164 | 2252 | 50 | 19 |
|  | F2 | 16179801 | 18626307 | 2447 | 68 | 20 |
| **Cat II** | A2 | 24023216 | 26006108 | 1983 | 51 | 21 |
|  | A2 | 88709433 | 102069069 | 13360 | 320 | 22 |
|  | A2 | 110044529 | 112388961 | 2344 | 58 | 23 |
|  | A3 | 24891837 | 25176827 | 285 | 73 | 24 |
|  | A3 | 123541136 | 130087880 | 6547 | 157 | 25 |
|  | B2 | 124384731 | 133037426 | 8653 | 234 | 26 |
|  | B3 | 24340297 | 30901705 | 6561 | 53 | 27 |
|  | B3 | 35478417 | 39267380 | 3789 | 83 | 28 |
|  | B3 | 101617843 | 103520805 | 1903 | 54 | 29 |
|  | B4 | 44637758 | 49171388 | 4534 | 123 | 30 |
|  | B4 | 74387753 | 82093062 | 7705 | 193 | 31 |
|  | B4 | 87057307 | 90420045 | 3363 | 88 | 32 |
|  | B4 | 92189979 | 102727651 | 10538 | 252 | 33 |
|  | C1 | 5901616 | 11173627 | 5272 | 127 | 34 |
|  | C1 | 133082713 | 135310424 | 2228 | 60 | 35 |
|  | C1 | 136106566 | 159953150 | 23847 | 593 | 36 |
|  | C2 | 10523779 | 12232391 | 1709 | 51 | 37 |
|  | D1 | 33532235 | 36915195 | 3383 | 72 | 38 |
|  | D1 | 48094697 | 53551934 | 5457 | 141 | 39 |
|  | D2 | 17623092 | 20597165 | 2974 | 67 | 40 |
|  | D2 | 62661535 | 64913164 | 2252 | 50 | 41 |
|  | D3 | 44573626 | 47918229 | 3345 | 83 | 42 |
|  | D4 | 11766310 | 18296932 | 6531 | 163 | 43 |
|  | F1 | 14553822 | 16744142 | 2190 | 48 | 44 |
|  | F1 | 42404967 | 55825177 | 13420 | 287 | 45 |
|  | F2 | 15324401 | 18310502 | 2986 | 85 | 46 |
|  | A1 | 52875446 | 66358246 | 13483 | 342 | 47 |
| **Cat III** | A2 | 23346211 | 34455598 | 11109 | 273 | 48 |
|  | A2 | 132969906 | 135135924 | 2166 | 62 | 49 |
|  | A3 | 95988947 | 98095833 | 2107 | 47 | 50 |
|  | A3 | 123541136 | 130087880 | 6547 | 157 | 51 |
|  | A3 | 107486123 | 110350037 | 2864 | 69 | 52 |
|  | B1 | 42175163 | 44812023 | 2637 | 63 | 53 |
|  | B1 | 47402716 | 52089988 | 4687 | 126 | 54 |
|  | B1 | 77872594 | 85777730 | 7905 | 198 | 55 |
|  | B1 | 115077126 | 116856658 | 1780 | 55 | 56 |
|  | B1 | 117098809 | 123430578 | 6332 | 164 | 57 |
|  | B3 | 13221030 | 35525404 | 22304 | 516 | 58 |
|  | B3 | 79902145 | 82752371 | 2850 | 63 | 59 |
|  | B3 | 90169790 | 98836662 | 8667 | 211 | 60 |
|  | B3 | 111251352 | 114015111 | 2764 | 68 | 61 |
|  | B4 | 79373420 | 90844601 | 11471 | 277 | 62 |
|  | C1 | 131544554 | 134065386 | 2521 | 57 | 63 |
|  | C1 | 194960094 | 197659658 | 2700 | 74 | 64 |
|  | C2 | 54946161 | 68256349 | 13310 | 52 | 65 |
|  | C2 | 126406897 | 128209705 | 1803 | 48 | 66 |
|  | D1 | 66524357 | 68761582 | 2237 | 65 | 67 |
|  | E1 | 24701530 | 53553890 | 28852 | 66 | 68 |
|  | E2 | 7657612 | 10800263 | 3143 | 84 | 69 |
|  | E2 | 41189203 | 44824568 | 3635 | 92 | 70 |
|  | E3 | 4317521 | 8818006 | 4500 | 113 | 71 |
|  | E3 | 18519294 | 20460379 | 1941 | 49 | 72 |
|  | F1 | 43117672 | 58219042 | 15101 | 337 | 73 |

^*^Position based on cat reference v8.0.

**Table 7.** RoH identified with WGS data on the three cats. The last column has the overlap with SNPchip RoH number.

|  | **Chr** | **Start pos.** ^*^ | **End pos.** ^*^ | **Length in KB** | **# of SNPs** | **Overlap** |
| --- | --- | --- | --- | --- | --- | --- |
| **Cat I** | A1 | 101015903 | 101273625 | 257.722 | 1101 |  |
|  | A1 | 185613324 | 185874535 | 261.211 | 521 |  |
|  | A2 | 167637890 | 167917112 | 279.222 | 1419 |  |
|  | A3 | 31987430 | 32264606 | 277.176 | 1168 | 4 |
|  | A3 | 32464471 | 33121711 | 657.24 | 1097 | 4 |
|  | A3 | 58341099 | 58594197 | 253.098 | 664 |  |
|  | B1 | 148265884 | 148584742 | 318.858 | 366 | 7 |
|  | B1 | 148788785 | 149044079 | 255.294 | 1129 | 7 |
|  | B1 | 150668423 | 150939220 | 270.797 | 724 | 7 |
|  | B1 | 151098350 | 151443157 | 344.807 | 763 | 7 |
|  | B1 | 151652866 | 151991363 | 338.497 | 1014 | 7 |
|  | B1 | 155771815 | 156121774 | 349.959 | 1391 | 7 |
|  | B1 | 156699503 | 157039874 | 340.371 | 1313 | 7 |
|  | B1 | 160222733 | 160560984 | 338.251 | 1310 | 8 |
|  | B4 | 23271966 | 23522123 | 250.157 | 925 | 10 |
|  | B4 | 25761438 | 26032550 | 271.112 | 1462 | 10 |
|  | C1 | 74806199 | 75065033 | 258.834 | 882 | 13 |
|  | C1 | 76572201 | 76885042 | 312.841 | 643 | 13 |
|  | C1 | 81828891 | 82088951 | 260.06 | 499 | 13 |
|  | C1 | 82885469 | 83396139 | 510.67 | 1072 | 13 |
|  | C1 | 83808436 | 84063716 | 255.28 | 708 | 13 |
|  | C1 | 84477764 | 84799796 | 322.032 | 1334 | 13 |
|  | C1 | 103888553 | 104155014 | 266.461 | 1177 | 14 |
|  | C1 | 113436413 | 113763049 | 326.636 | 1486 | 14 |
| **Cat II** | A2 | 70317671 | 70587500 | 269.829 | 1401 |  |
|  | A2 | 93420540 | 93738153 | 317.613 | 763 | 22 |
|  | A2 | 100350902 | 100684673 | 333.771 | 1628 | 22 |
|  | A2 | 139834123 | 140119510 | 285.387 | 1584 |  |
|  | A3 | 6767689 | 7044783 | 277.094 | 1694 |  |
|  | A3 | 127938965 | 128256244 | 317.279 | 1864 | 25 |
|  | B1 | 79235467 | 79690261 | 454.794 | 993 |  |
|  | B2 | 114382954 | 114635459 | 252.505 | 530 |  |
|  | B2 | 126837263 | 127146173 | 308.91 | 1013 | 26 |
|  | B2 | 130081082 | 130441813 | 360.731 | 1030 | 26 |
|  | B2 | 131188885 | 131474676 | 285.791 | 1238 |  |
|  | B4 | 76332649 | 76752295 | 419.646 | 1823 | 31 |
|  | B4 | 80570741 | 80889792 | 319.051 | 1415 | 31 |
|  | B4 | 98500798 | 98772486 | 271.688 | 1731 | 33 |
|  | B4 | 135465338 | 135737942 | 272.604 | 862 |  |
|  | C1 | 141575297 | 141837677 | 262.38 | 796 | 36 |
|  | C1 | 144412609 | 144810398 | 397.789 | 306 | 36 |
|  | C1 | 149983559 | 150249963 | 266.404 | 552 | 36 |
|  | C1 | 152195815 | 152558284 | 362.469 | 1147 | 36 |
|  | D1 | 52422798 | 52877160 | 454.362 | 2082 | 39 |
|  | D2 | 16292437 | 16606291 | 313.854 | 2134 |  |
|  | D2 | 16606376 | 16894580 | 288.204 | 1108 |  |
|  | F1 | 48416341 | 48728996 | 312.655 | 1080 | 45 |
|  | F1 | 48986161 | 49465663 | 479.502 | 1742 | 45 |
|  | F1 | 55253519 | 55523100 | 269.581 | 1082 | 45 |
|  | F1 | 63134633 | 63405128 | 270.495 | 1303 |  |
|  | F2 | 23895040 | 24157690 | 262.65 | 1400 |  |
| **Cat III** | A1 | 55327438 | 55749293 | 421.855 | 1905 | 47 |
|  | A1 | 57497935 | 57751689 | 253.754 | 874 | 47 |
|  | A1 | 58536117 | 58946873 | 410.756 | 1324 | 47 |
|  | A1 | 61991104 | 62424144 | 433.04 | 1585 | 47 |
|  | A1 | 65406379 | 65799827 | 393.448 | 2049 | 47 |
|  | A2 | 23634415 | 23957925 | 323.51 | 1163 | 48 |
|  | A2 | 24955187 | 25244773 | 289.586 | 819 | 48 |
|  | A2 | 26304226 | 26580999 | 276.773 | 772 | 48 |
|  | A2 | 27075215 | 27397518 | 322.303 | 1164 | 48 |
|  | A2 | 28554310 | 28819362 | 265.052 | 1388 | 48 |
|  | A2 | 29638551 | 29970801 | 332.25 | 1826 | 48 |
|  | A2 | 31415774 | 31694723 | 278.949 | 1055 | 48 |
|  | A2 | 48107026 | 48364277 | 257.251 | 1685 |  |
|  | A3 | 124910332 | 125219685 | 309.353 | 683 | 51 |
|  | A3 | 125756085 | 126027529 | 271.444 | 854 | 51 |
|  | B1 | 50399977 | 50665058 | 265.081 | 1290 | 54 |
|  | B1 | 78319317 | 78593917 | 274.6 | 1264 | 55 |
|  | B1 | 81153778 | 81627423 | 473.645 | 2298 | 55 |
|  | B1 | 82619073 | 83033383 | 414.31 | 1202 | 55 |
|  | B1 | 84470384 | 84952987 | 482.603 | 2546 | 55 |
|  | B1 | 115185919 | 115511141 | 325.222 | 1346 | 56 |
|  | B1 | 115837409 | 116130772 | 293.363 | 1658 | 56 |
|  | B1 | 117346186 | 117669575 | 323.389 | 1864 | 57 |
|  | B1 | 118305159 | 118618701 | 313.542 | 1470 | 57 |
|  | B3 | 16102154 | 16381224 | 279.07 | 1498 | 58 |
|  | B3 | 16846546 | 17143829 | 297.283 | 1968 | 58 |
|  | B3 | 17415555 | 17708778 | 293.223 | 1334 | 58 |
|  | B3 | 17798942 | 18242834 | 443.892 | 2194 | 58 |
|  | B3 | 18405748 | 18721739 | 315.991 | 1403 | 58 |
|  | B3 | 21875907 | 22265139 | 389.232 | 1158 | 58 |
|  | B3 | 22516772 | 22894439 | 377.667 | 1848 | 58 |
|  | B3 | 23119013 | 23456285 | 337.272 | 736 | 58 |
|  | B3 | 24240443 | 24567376 | 326.933 | 528 | 58 |
|  | B3 | 25110150 | 25396170 | 286.02 | 346 | 58 |
|  | B3 | 30343421 | 30654326 | 310.905 | 245 | 58 |
|  | B3 | 93244704 | 93572575 | 327.871 | 1175 | 60 |
|  | B3 | 94949079 | 95248812 | 299.733 | 1160 | 60 |
|  | B4 | 84481884 | 84747441 | 265.557 | 896 | 62 |
|  | B4 | 84840697 | 85216737 | 376.04 | 1283 | 62 |
|  | C1 | 76507030 | 76819618 | 312.588 | 876 |  |
|  | C1 | 133578908 | 133831526 | 252.618 | 342 | 63 |
|  | C2 | 123733610 | 124080254 | 346.644 | 1842 |  |
|  | D2 | 7355114 | 7641797 | 286.683 | 2056 |  |
|  | D2 | 63920539 | 64195251 | 274.712 | 757 |  |
|  | E2 | 41473036 | 41740866 | 267.83 | 1418 | 70 |
|  | E2 | 43447773 | 43789212 | 341.439 | 883 | 70 |
|  | E2 | 44345775 | 44598136 | 252.361 | 680 | 70 |
|  | E3 | 40605471 | 40889292 | 283.821 | 268 |  |
|  | F1 | 43666028 | 43940574 | 274.546 | 1406 | 73 |
|  | F1 | 44215646 | 44539911 | 324.265 | 1534 | 73 |
|  | F1 | 46333558 | 46634075 | 300.517 | 1209 | 73 |
|  | F1 | 48984963 | 49465663 | 480.7 | 1744 | 73 |
|  | F1 | 51179504 | 51490114 | 310.61 | 1075 | 73 |
|  | F1 | 54130303 | 54613982 | 483.679 | 2146 | 73 |
|  | F1 | 54665695 | 55065895 | 400.2 | 1600 | 73 |
|  | F1 | 56830780 | 57240287 | 409.507 | 1155 | 73 |
|  | F2 | 45803411 | 46069477 | 266.066 | 908 |  |

^*^Position based on cat reference v8.0.
